# Relationship between SIRT1 gene expression level and disease in age-related cataract cases

**DOI:** 10.3906/sag-1810-182

**Published:** 2019-08-08

**Authors:** Nihat Buğra AĞAOĞLU, Nuray VAROL, Saliha Handan YILDIZ, Cem KARAOSMANOĞLU, Rahmi DUMAN, Müjgan ÖZDEMİR ERDOĞAN, Mustafa SOLAK

**Affiliations:** 1 Department of Medical Genetics, Umraniye Training and Research Hospital, İstanbul Turkey; 2 Department of Medical Genetics, Afyonkarahisar Health Sciences University, Afyonkarahisar Turkey; 3 Department of Eye Diseases, LIV Hospital, Ankara Turkey; 4 Department of Medical Biology, Afyonkarahisar Health Sciences University, Afyonkarahisar Turkey

**Keywords:** Age-related cataract, epigenetics mechanisms, SIRT1

## Abstract

**Background/aim:**

Age-related cataract is the most important visual impairment all over the world. Epigenetic modifications, especially overexpression of histone deacetylases, have become the focus of interest for cataract development in recent years. Sirtuin 1 (SIRT1), a class II histone deacetylase and a member of the sirtuin family, is one of the best-characterized histone deacetylases and has a pivotal role in age-related diseases. However, the association of SIRT1 with age-related cataracts has not yet been fully elucidated. Therefore, we aimed to determine the expression of* SIRT1 *in age-related cataract patients.

**Materials and methods:**

Expressions of *SIRT1* were evaluated by quantitative polymerase chain reaction (qPCR) in patients and healthy controls. RNA samples were collected from the anterior capsule and peripheral blood samples of age-related cataract patients. Human lens epithelial cell line B3 and peripheral blood samples of healthy subjects were used as controls.

**Results:**

We determined that the expression of *SIRT1* in blood and anterior capsule samples increased significantly compared to the control group (P < 0.05).

**Conclusion:**

The expression level of SIRT1 plays a vital role in the development of age-related cataract and it can be used as a biomarker. Thus, *SIRT1* inhibitors can be used in the treatment of age-related cataract disease.

## 1. Introduction

A Cataract is a blurring of the natural lens in the eye. Although the main cause of cataract is aging, trauma can occur due to toxic causes, systemic disorders (e.g., diabetes), smoking, and hereditary factors [1]. Age-related cataract is the most common cause of vision loss. Studies showed that the incidence of cataract was 50% in the 65–74 age group and more than 70% in those over 75 years [2]. Despite the complex structure of the lens, preservation of transparency throughout life requires the elimination of many negative factors. Although there are many causes of opacification of the lens fibers, the first and most important cause is aging [1]. The development of cataract depends on many factors and it is usually characterized by deterioration of the lens microstructure. Age-related cataracts develop as a result of interaction between genes and environmental factors that affect the phenotype [3]. 

Sirtuin 1 (SIRT1) is a class III NAD+-dependent protein deacetylase and a member of the sirtuin family. SIRT1 protein is in both the nucleus and the cytoplasm. SIRT1 has a pivotal role in cancer development, programmed cell death, regulation of gene expression, DNA repair, and aging mechanisms. SIRT1 controls energy homeostasis during cellular stress. When SIRT1-mediated energy homeostasis is not achieved, it promotes cellular aging to avoid genomic instability. Sirtuins play a pivotal role in maintaining cellular homeostasis by the regulation of both epigenetic and non-epigenetic mechanisms. SIRT1 was shown to be associated with various age-related and ocular diseases. It can also play an important role in the process of self-renewal and aging of ocular stem cells [4]. Aging is systematically associated with sirtuin-induced mitochondrial DNA instability. Briefly, SIRT1 regulates DNA stability and ensures the survival of the cell [5–8].

SIRT1 is important for eye development. Retinal cells are preserved from DNA damage through oxidative stress, apoptotic retinal death, and anti-inflammation. SIRT1 also has a protective role in neurons in cases of retinal damage. Moreover, SIRT1 fragmentation induces retinal damage with various mechanisms. First of all, it was shown that expressions of the *Oct4 *and* SIRT1* genes were reduced in older retinal pigment epithelial cells or slightly injured rat retinas. Secondly, retinal activator protein-1 was overexpressed and retinal SIRT1 activity was decreased in mice with light exposure. Finally, retinal inflammation was induced with downregulation of the adenosine monophosphate (AMP) activated protein kinase (AMPK) pathway and NF-κB activation associated with SIRT1 deactivation in diabetes. Cellular energy homeostasis alteration causes oxidative stress. SIRT1 maintains energy homeostasis and antiapoptotic mechanisms that are necessary for optimal normal brain function in the normal adult central nervous system for the regulation of oxidative stress [4]. 

Moreover, SIRT1 is believed to contribute to the protection of neurons from Wallerian degeneration according to other studies [9,10]. Therefore, in this study, we evaluated the expression of SIRT1 in the lens epithelium and peripheral blood samples in age-related cataract patients.

## 2. Materials and methods

### 2.1. Patient selection 

This prospective single-center study was carried out with 100 cataract patients (age range: 40–90 years, 55 men and 45 women) and 100 healthy controls (age range: 39–83 years) admitted to the Ophthalmology Clinic of Kocatepe University Hospital (Afyonkarahisar, Turkey) from October 2016 to December 2017. The selection criteria for participants were: diagnosis with age-related cataract, having visual impairment due to cataract, and age ≥40 years and <90 years. Exclusion criteria were a history of intraocular surgery, ocular inflammation, diabetes, ocular trauma, using systemic or topical steroids, receiving eye radiotherapy, and drug use with congenital cataract toxic cataracts (phenothiazines, cholinergics, cancer drugs, photosensitive drugs, diuretics, tranquilizers, and gut mediators).

### 2.2. Cell lines and primer culture

The human lens epithelial cell line (HLE-B3) was provided from the American Type Culture Collection (ATCC). Cells were maintained in Eagle’s minimum essential medium (Sigma-Aldrich, USA) supplemented with 20% fetal bovine serum (FBS) (Sigma-Aldrich). 

Ex vivo growth of anterior capsules including lens epithelial cells from cataract patients were previously described [11]. Anterior capsule specimens of approximately 5 mm were shaken down in 1.5-mL tubes containing medium supplemented with 10% FBS and 1% penicillin/streptomycin (solution stabilized with 10,000 units of penicillin and 10 mg/mL streptomycin) after the surgery. Anterior capsules were transferred to 24-well culture dishes containing standard culture medium (RPMI-1640) including 10% FBS. The capsules were allowed to settle freely at the bottom of the cell culture wells and were grown to confluence in 24-well culture dishes. The culture dishes were subsequently put into a humidified CO2 incubator at 37 °C to allow the capsules to attach to the bottom of the culture wells. Capsules were passaged after growing to 80% confluency and replaced equally (1 × 106 cells/mL) into sterile 35-cm2 petri dishes for total RNA extraction.

### 2.3. Quantitative real-time PCR 

Total RNA was extracted using QIAzol reagent (QIAGEN, Germany). Total RNA (1 µg) was reverse-transcribed using the RT2 HT First Strand Kit (QIAGEN) according to the manufacturer’s instructions. *SIRT1 *mRNA expression level was measured by the quantitative real-time PCR method (qPCR). qPCR was performed on the RotorGene Q using qPCR SYBR Green Master Mix (QIAGEN) and the *SIRT1* RT2 primer assay (NM_001142498, Cat No. PPH02188A, QIAGEN). Gene expression levels were normalized to *GAPDH*. 

### 2.4. Statistical analysis

The results of cDNA synthesis and qPCR were replicated twice, separately. Statistical significance levels of mRNA expressions were analyzed using the pairwise fixed reallocation randomization test. REST software (2009) was used for group-wise comparisons and statistical analysis of the expression levels.

## 3. Results

### 3.1. Evaluation of the expression levels of SIRT1 genes

**Figure 1:**
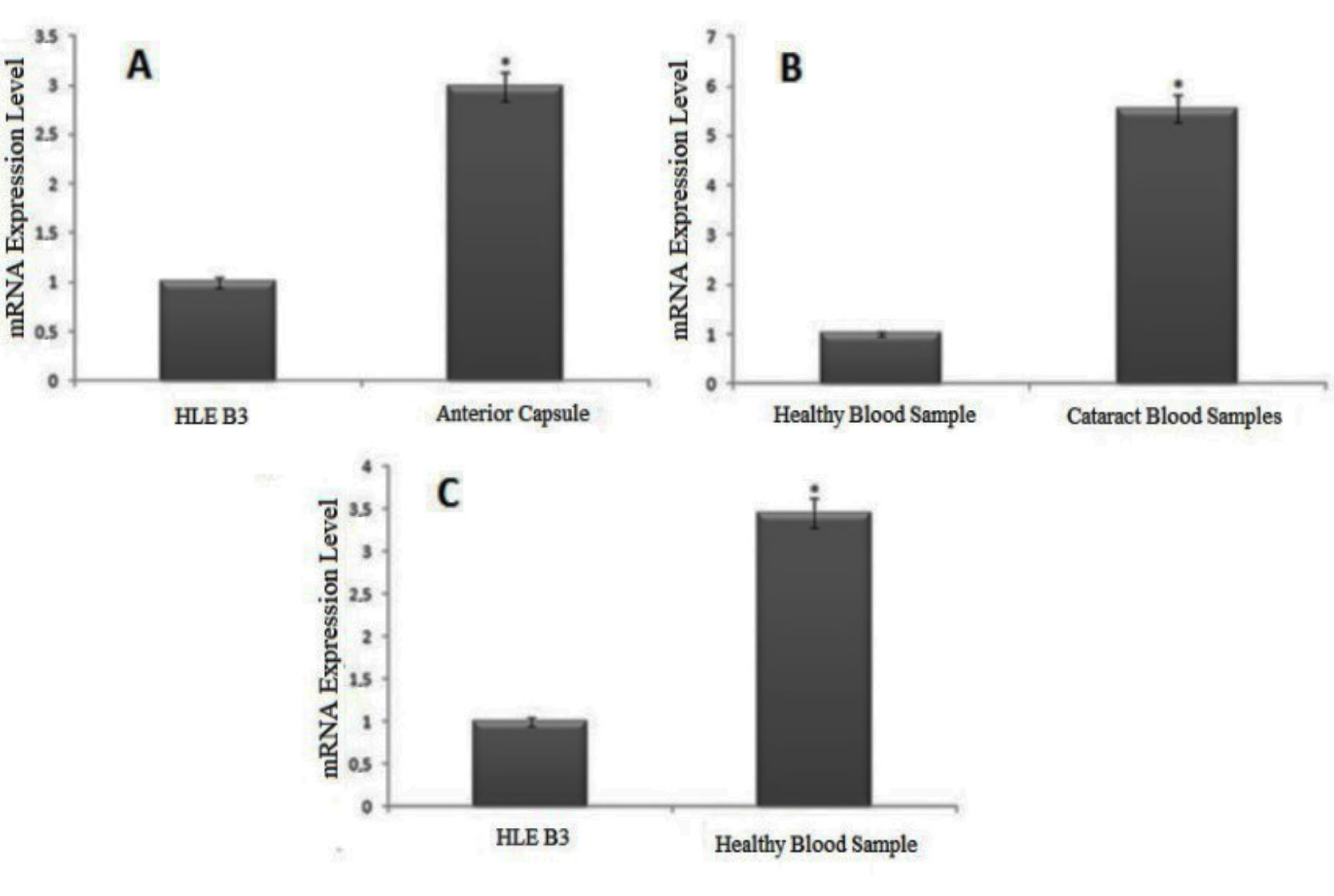
The Expression level of SIRT1 in anterior capsule samples compared to HLE B3 cell line (A), in peripheral blood samples compared to healthy blood samples (B), and in HLE B3 compared to healthy blood samples (C). Expression levels of target genes were normalized based on the GAPDH mRNA expression level. *: p< 0.05.

The expression levels of *SIRT1 *were examined using qPCR. In the 75 anterior capsule samples, the mRNA level of the *SIRT1* gene was increased by 2.99-fold compared to normal human lens epithelial cells (HLE B3 cell line) (P < 0.05) (Figure 1a). In 80 peripheral blood samples, the mRNA level of the *SIRT1* gene was increased by 5.54-fold compared to the blood samples of healthy individuals (P < 0.05) (Figure 1b).

We observed a 5.54-fold increase comparing blood samples from cataract patients with control samples of healthy individuals. To understand the reason for this difference between anterior capsules and blood samples, the mRNA levels of the* SIRT1* gene in the blood samples of healthy individuals were increased by 2.99 times compared with the HLE B3 cell line (P < 0.05) (Figure 1c).

**Figure 2:**
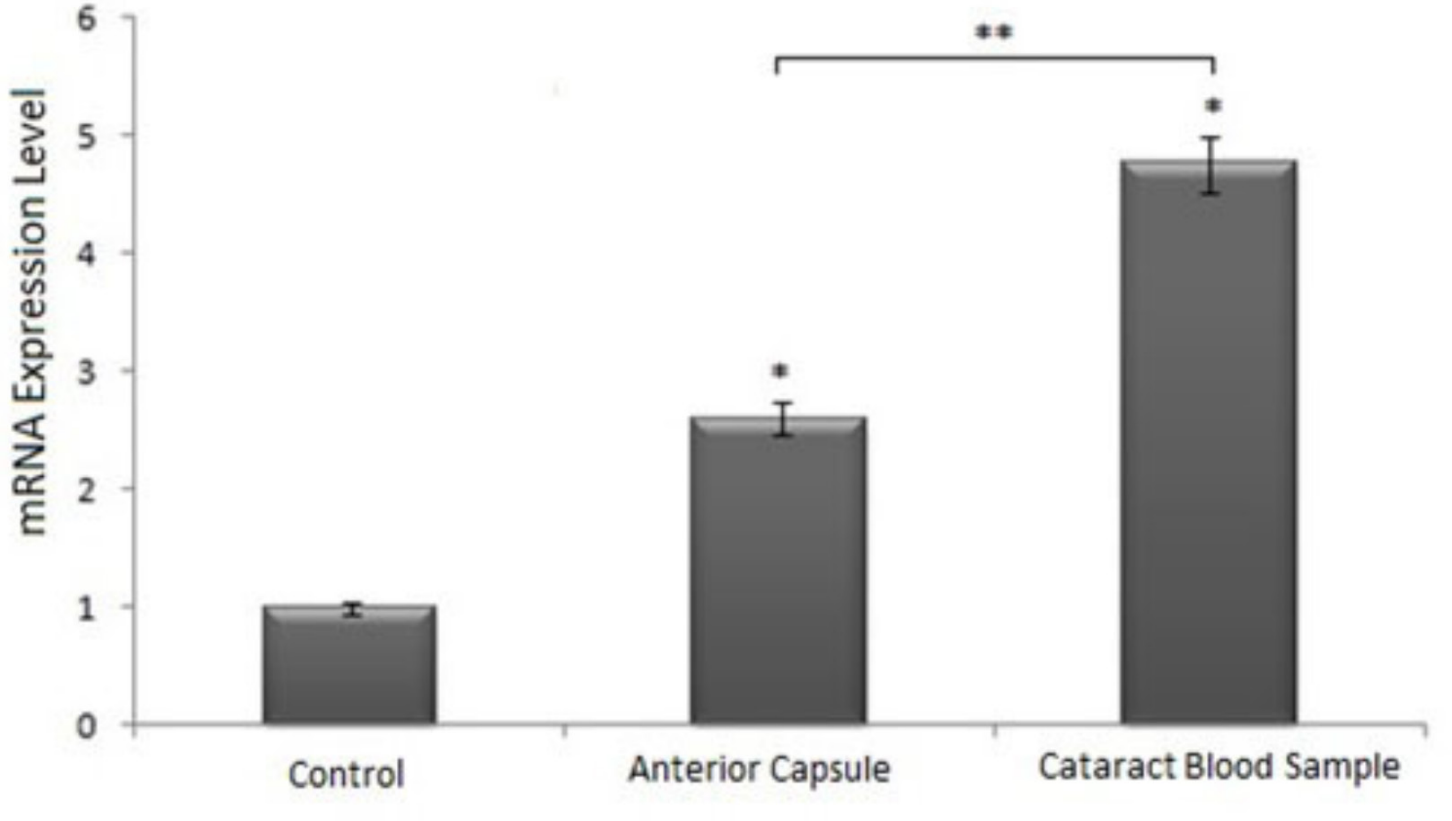
Changes in SIRT1 mRNA levels in anterior capsules and peripheral blood samples of cataract patients. Expression levels of target genes were normalized based on the expression level of GAPDH mRNA. * and **: p < 0.05.

When the anterior capsule samples and peripheral blood samples of randomly selected cataract patients were compared with HLE B3 cells, we observed a 2.60-fold increase in the *SIRT1* mRNA level in the anterior capsule sample, and the increase in the peripheral blood sample of the same patient was 16.68-fold (P < 0.05). When the peripheral blood sample was compared with the blood sample of a healthy individual, a 4.76-fold increase was observed. This shows that there was a statistically significant increase in the expression level of the *SIRT1* gene in both anterior capsule and peripheral blood samples. However, the expression level of the *SIRT1* gene was significantly higher in peripheral blood samples than the anterior capsule (P < 0.05) (Figure 2). 

## 4. Discussion

There has been a significant increase in the average lifespan due to industrialization. Aging problems arise as a result of the increase in average life expectancy [12]. One of the most common age-related problems is the loss of vision due to cataract. Although there are many factors in its etiology, the mechanism of cataract development is not exactly known [13]. Physiological status, environmental components, and genetic differences of age-related cataract indicate that it is a multifactorial disease [14,15]. 

Sirtuins are class III histone deacetylases that control energy homeostasis in response to stress and promote cellular aging to avoid genomic instability when homeostasis fails [16]. SIRT1 has been shown to be associated with ocular diseases such as cataract, retinal degeneration, optic neuritis, and uveitis [4]. Besides these functions, a pivotal role of SIRT1 in the eye may be to protect the retina and optic nerve from degeneration. Peng et al. found that SIRT1 expression and self-renewal capacity are decreased in rats and humans with aging [17]. This indicates that SIRT1 plays an effective role in anti-aging and protection against neurodegenerative disorders when calorie restriction is used. In this study, we examined the changes in mRNA levels of the *SIRT1* gene in anterior capsules and peripheral blood samples were taken from patients undergoing cataract surgery. Advanced-aged patients with cataracts were also included. When anterior capsule samples of advanced-aged cataract patients were compared with HLE B3 cells, expression of the *SIRT1* gene was significantly increased in almost all samples (P < 0.05). Similarly, peripheral blood samples of patients with cataract were found to be statistically significant when compared to peripheral blood samples of healthy individuals (P < 0.05). In the study of Jaliffia et al., the SIRT1 protein was found to be localized in the adult mouse lens epithelium and fiber cell nuclei, but not in the lens capsule [18].

In the human eye, expression of the *SIRT1* gene was detected in the lens epithelium [12,19] and in the retina [20] of patients with senile cataract. In normal human corneal epithelium, Alves et al. reported negative *SIRT1 *expression in 50%, weak expression in 30%, and significant immunoreactive expression in 20% using 10 corneal specimens [21]. In accordance with our study, Zheng and Lu used anterior lens capsules in their study [12]. In this study, capsule samples without cataract from patients of less than 49 years of age were compared to capsule samples without cataract from those over 50 years of age and a significant decrease in the mRNA level of the *SIRT1* gene was observed. When the anterior capsule samples from those with cataract over 50 years of age were compared with anterior capsule samples from the same age group without cataract, it was determined that the expression level of the *SIRT1* gene was significantly increased [12]. There are 9 exons of the *SIRT1* gene and 6 alternative splicing variants, and only four of these variants encode the protein. The *SIRT1* primers we used in our study were designed to be specific to common regions in these variants.

Furthermore, Zheng et al. showed that *SIRT1* expression in the lens epithelium decreased with age, but there was an increase in cataract patients over 50 years old compared to the control group [12]. Similarly, in our study, we found a significant increase in the expression level of the *SIRT1* gene in the older cataract patients.

In conclusion, SIRT1 is an important histone deacetylase and has a wide variety of important roles in maintaining cellular homeostasis. It is an attractive candidate for the development of therapeutic strategies for the prevention of eye diseases associated with aging, especially cataracts. Some clinical studies for SIRT1 activators have already been initiated for diseases including cardiovascular disease, human cancers, diabetes, and Alzheimer disease. Future clinical studies should focus on the precise definition of SIRT1’s role in eye aging.

## Acknowledgment

This project was supported by the Afyon Kocatepe University Board of Scientific Research Projects, Afyonkarahisar, Turkey, Project Number 16.SAĞ.BİL.26.

## References

[ref0] (2016). Common eye diseases and their management. Cataract.

[ref1] (2011). Vaughan & Asbury’s General Ophthalmology.

[ref2] (2011). -2012 Basic and clinical science course, section 11: Lens and cataract. Pathology. Revised Edition.

[ref3] (2013). The role of SIRT1 in ocular aging. Experimental Eye Research.

[ref4] (2004). Stress-dependent regulation of FOXO transcription factors by the SIRT1 deacetylase.

[ref5] (2004). Hekking B et al. Science.

[ref6] (2007). SIRT1 regulates the function of the Nijmegen breakage syndrome protein. Molecular Cell.

[ref7] (2010). Dual role of Zn2+ in maintaining structural integrity and suppressing deacetylase activity of SIRT1. Journal of Inorganic Biochemistry.

[ref8] (2008). Chua CE. SIRT1 and neuronal diseases. Molecular Aspects of Medicine.

[ref9] (2010). SIRT1-dependent regulation of chromatin and transcription: linking NAD+ metabolism and signaling to the control of cellular functions. Biochimica et Biophysica Acta.

[ref10] (2014). In vitro growth of lens epithelial cells from cataract patients-association with possible risk factors for posterior capsule opacification. Open Ophthalmology Journal.

[ref11] (2011). Changes in SIRT1 expression and its downstream pathways in age-related cataract in humans.

[ref12] (2014). Etiopathogenesis of cataract: an appraisal. Indian Journal of Ophthalmology.

[ref13] (1995). Epidemiology of risk factors for age-related cataract. Survey of Ophthalmology.

[ref14] (2011). Aboukhalil A et al. Science.

[ref15] (2010). Fraga MF. Discovery Medicine.

[ref16] (2010). Wu CC et al. Sensors.

[ref17] (2009). Vieira V et al. Investigative Ophthalmology & Visual Science.

[ref18] (2011). Lin-Chung-Woung et al. Journal of Cataract & Refractive Surgery.

[ref19] (2012). Doyle M et al. Stem Cells International.

[ref20] (2012). Maloney S et al. Cornea.

